# Trends and factors associated with delays in the first prenatal consultation in Guinea

**DOI:** 10.4102/jphia.v16i1.502

**Published:** 2025-01-30

**Authors:** Tiany Sidibe, Mamadou Dioulde Balde, Sadan Camara, Ramata Diallo, Bienvenu Salim Camara, Karifa Kourouma, Madeleine Toure, Kaba Saran Keita, Fanta Barry, Maimouna Balde

**Affiliations:** 1 Department of Public Health, Center for Research in Reproductive Health in Guinea, Conakry, Guinea; 2 Department of Public Health, Faculty of Health Sciences and Techniques, Gamal Abdel Nasser University of Conakry, Conakry, Guinea

**Keywords:** trends, associated factors, delay in first antenatal care, Guinea, DHS

## Abstract

**Background:**

The first antenatal care (ANC1) is considered late if it is performed after the first 12 weeks of pregnancy. In Guinea, this phenomenon remains under-analysed.

**Aim:**

The objective of this study is to analyse the trends and factors associated with the delay in performing the ANC1 in Guinea from 2007 to 2018.

**Setting**: This study was conducted in Guinea.

**Methods:**

A secondary analysis of the 2012 and 2018 Demographic and Health Surveys in Guinea was conducted. The study included women aged 15-49 years who gave birth in the five years prior to the surveys and had at least one ANC visit for their last child. Multivariate logistic regression was used to identify associated factors using Stata 17.

**Results:**

This study shows that in Guinea, out of 14 546 women, the overall proportion of the delay in performing the ANC1 between 2007 and 2018 was 73%. The trend of this proportion decreased from 86% in 2007 to 61% in 2010, from 85% in 2013 to 61% in 2016; however, it increased from 61% in 2010 to 85% in 2013 and from 66% in 2016 to 76% in 2018. The factors associated with the delay in performing the ANC1 were: being aged 35–49 years (adjusted odds ratio [AOR]: 1.36; 95% confidence interval [CI]: 1.08–1.69); Living in a poor household (AOR: 1.87; 95% CI: 1.64–2.13), living in Boké (AOR: 2.13; 95% CI: 1.58–2.87), and N’zerekore (AOR: 4.97; 95% CI: 3.58–6.91).

**Conclusion:**

We recommend stepping up door-to-door awareness-raising activities by community relays and ensuring that the policy of free antenatal care in Guinea is effective.

**Contribution:**

This study shows a very high prevalence of delay in the ANC1 in Guinea influenced by many factors.

## Introduction

The antenatal care (ANC) is a key element for the health of mothers and their children, by providing essential care and recommendations for a healthy pregnancy.^1^ According to the World Health Organization (WHO) guidelines issued in 2016, it is advisable to make at least eight contacts in order to reduce the perinatal mortality rate and ensure quality care.^1^ However, the WHO emphasises that the first ANC (ANC1) is considered late if it is performed after the first 12 weeks of pregnancy.^1^ This delay in performing the ANC1 can deprive the pregnant woman of essential health interventions likely to prevent complications and adverse pregnancy outcomes.^2^ For example, in pregnant women with untreated early syphilis, the delay in performing the ANC1 beyond the 1st quarter of pregnancy can result in 80% of pregnancies with severe and adverse outcomes, such as stillbirth, premature delivery, neonatal death or congenital syphilis in the infant.^3,4^ In addition, the delay in performing the ANC1 plays a significant role in increasing perinatal complications such as low birth weight as well as maternal and neonatal deaths.^5,6^

In sub-Saharan Africa, studies have shown a high proportion of the delay in performing the ANC1. In Ethiopia, a study reported a prevalence of 80%^7^; in Uganda in 2022, it was 63.9%^8^ and 73.6% in Nigeria in 2021.^9^ On the other hand, in 2022 in Ethiopia, a study reported that the prevalence of the delay in performing the ANC1 decreased from 76.8% in 2000 to 67.3% in 2016.^10^

The literature reports that factors such as rural residence, level of education, distance from a health facility, low income, unplanned pregnancy and obstetric history as factors associated with the delay in performing the ANC1.^7,11,12^ In addition, caesarean deliveries, the family size and the desire to have no more children have also been cited by other studies as factors related to the delay in performing the ANC1.^7,10^

Since 2011, Guinea has adopted a policy of free maternal health care, including ANC.^13^ Despite this context of free care, in 2018, according to the recent report of the Demographic and Health Survey (DHS) in Guinea, only 35% of women aged from 15 years to 49 years attended the recommended four or more ANC. In addition, less than one in three women (only 29% of pregnant women) had their ANC1 on time, that is, during the first quarter of pregnancy.^14^

Although various studies have focussed on ANC in Guinea,^15,16,17,18,19^ little remains documented on the phenomenon of the delay in performing the ANC1 knowing that the timely ANC has a positive effect in preventing pregnancy-related complications and maternal mortality, which remains high in Guinea, at 576 maternal deaths per 100 000 live births in 2017.^14^

This study, therefore, aims to answer the following question: *What are the trends and factors associated with the delay in performing the ANC1 in Guinea, using data from the 2012 and 2018 DHSs?* The answer to this question will help to better guide policies and practices for the adequate use of ANC services in Guinea. The objective of this study was to analyse the annual trends and factors associated with the delay in performing ANC1 in Guinea from 2007 to 2018.

## Conceptual framework for delay in achieving first antenatal care

**FIGURE 1:**
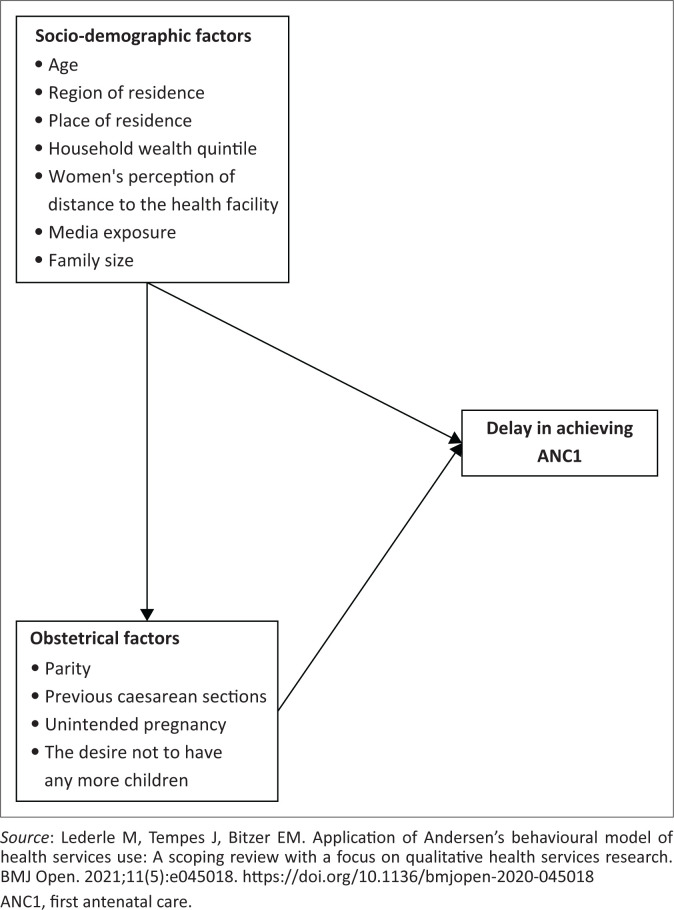
Conceptual framework of delay in achieving first antenatal care visit; adapted from Anderson’s behavioural model of health service use.

To identify the relevant variables available in the literature, we were inspired by Anderson’s behavioural model of health service use^20^ (Figure 1).^21^ We included the woman’s age at birth (15–24 years old; 25–34 years old; 35–49 years old), place of residence as specified in the DHS sampling strategy (rural, urban), region of residence (Conakry, Boké, Kindia, Mamou, Labé, Faranah, Kankan and Nzérékoré), the highest level of education of the woman at the time of the survey and of her spouse (no education level, primary and secondary or higher education), women’s perception of distance to the health facility (big problem or not), access to media (listening to radio or television or not), marital status of the woman at the time of the survey (married, single and divorced) and the wealth quintile which was grouped into three groups (poor, middle, rich), because of the very small sample size, were used as socio-demographic factors. For obstetric factors, the mother’s parity (primiparous, multiparous and grand multiparous), the desire for pregnancy (desired yes or no) and intentional pregnancy (yes or no) were used.

### Study framework

Guinea is located in West Africa and covers an area of 245 857 km^2^ with a population of over 13 million inhabitants, the majority of whom live in rural areas (63%).^22^ The country has 33 prefectures. The health system is based on a three-level pyramid. The primary level includes health posts and centres; the secondary level includes regional and prefectural hospitals, and communal medical centres; and the tertiary level includes three national hospitals. Prenatal care is provided at the primary and secondary levels. In 2014, the country had approximately two doctors and one nurse per 10 000 inhabitants, with a disparity between rural and urban areas.^22^

### Type and period of study

This is a secondary analysis of data from the 2012 and 2018 DHSs of Guinea, which are national cross-sectional household surveys.

### Study population

The study population consisted of women aged from 15 years to 49 years who had given birth in the 5 years preceding the two surveys and who had received at least the ANC1.

Knowing that the DHSs take into account data from the last 5 years preceding the survey, the periods 2007–2011 for the 2012 DHS and 2013–2017 for the 2018 DHS were considered to trace annual trends. Only information relating to the most recent pregnancy was considered.

### Study variables

The dependent variable of this study was the delay in performing the ANC1. This is a dichotomous variable, constructed as follows: all women who had benefitted from the ANC1 in the first quarter of pregnancy were grouped and coded as 0 and those who had their ANC1 after the first quarter of pregnancy were coded as 1.

The independent variables examined in this study were the age of the woman at birth, place of residence, region of residence, level of education of the woman and her spouse, women’s perception of distance from the health facility, access to media, marital status, wealth quintile, parity, desire for pregnancy, history of caesarean sections, intentional pregnancy and the profile of the agent who received the woman for the first time.

### Data analysis

Data were analysed using Stata software version 17.0 (StataCorp, College Station, Texas, United States [US]). For descriptive analysis, proportions with confidence intervals (CI) for qualitative variables and the mean with standard deviation (s.d.) for quantitative variables were calculated. Annual trends in the proportion of the delay in performing the ANC1 were presented as an epidemiological curve, and Pearson’s Chi-square test was used to compare them at the 5% threshold.

To determine the factors associated with the delay in performing the ANC1, univariate and multivariate logistic regression were performed after checking for multi-collinearity between independent variables. Crude and adjusted odds ratios (AOR) with 95% CI were calculated. To validate the final model, specification and adjustment tests were used (Linktest and LROC).

### Ethical considerations

A formal request for analysis of all data was made to MEASURE DHS via the online platform at https://dhsprogram.com/data/available-datasets.cfm. and permission was granted. The original data were collected with ethical approval from the National Health Research Ethics Committee of Guinea and the ICF International Review Board.

## Results

### Description of the study sample

A total of 14 546 women were included, 13 208 women (90.8%) were married, 11 332 had no education level (77.9%) and 10 052 lived in rural areas (69.1%) (Table 1). The average age of these women was 28 years (s.d.: 7.3 years). Nearly half of them were grand multiparous (48.4%).

**TABLE 1:** Socio-demographic and obstetric characteristics of women who underwent first antenatal care in Guinea, 2012 and 2018 Demographic and Health Surveys (***N*** = 14 546).

Variables	*n*	%	Mean	s.d.
**Age group (years)**	-	-	28	7.3
15–24	4790	33.6	-	-
25–34	5663	38.9	-	-
35–49	4092	27.5	-	-
**Region of residence**	-	-	-	-
Boké	1784	10.5	-	-
Conakry	1668	14.9	-	-
Faranah	1966	9.7	-	-
Kankan	1951	14.7	-	-
Kindia	1846	14.8	-	-
Labé	1773	10.4	-	-
Mamou	1681	8.2	-	-
N’zérékoré	1876	16.8	-	-
**Place of residence**	-	-	-	-
Urban	4678	30.9	-	-
Rural	9867	69.1	-	-
**Household wealth index**	-	-	-	-
Poor	6073	41.26	-	-
Intermediate	2955	21.25	-	-
Rich	5517	37.49	-	-
**Woman’s education level**	-	-	-	-
No formal education	11 357	77.9	-	-
Primary	1532	10.5	-	-
Secondary	1359	9.4	-	-
Higher	297	2.2	-	-
**Partner’s education level**	-	-	-	-
No formal education	9735	78.9	-	-
Primary	819	7.2	-	-
Secondary	328	2.8	-	-
Higher	1296	11.1	-	-
**Whether the woman had a job**	-	-	-	-
No	3640	24.4	-	-
Yes	10 899	75.6	-	-
**Decision making to go for antenatal care**	-	-	-	-
Woman alone	1206	9.9	-	-
Woman and her partner	3670	27.9	-	-
Partner alone	8264	62.2	-	-
**Distance to the health facility perceived as a problem**	-	-	-	-
Yes	3764	47.6	-	-
No	3922	52.4	-	-
**Marital status**	-	-	-	-
Single	637	4.8	-	-
Married	13 253	90.8	-	-
Divorced/widowed	655	4.4	-	-
**Profile of health worker who provided the first antenatal care**	-	-	-	-
Physician	1366	9.6	-	-
Midwife	5073	35.6	-	-
Technical health agent	1276	9.1	-	-
Other	6831	45.7	-	-
**Whether the pregnancy was intended**	-	-	-	-
Yes	8583	82.4	-	-
No	1815	17.6	-	-
**Parity**	-	-	-	-
Primiparous†	2553	17.9	-	-
Multiparous†	4799	33.5	-	-
Large multiparous†	7193	48.4	-	-
**History of caesarean section**	-	-	-	-
Yes	312	3.1	-	-
No	14 227	96.9	-	-

s.d., standard deviation.

† , Primiparous = 1 child; Multiparous = 2–4 children; Large multiparous = More than 4 children.

### Trends in the proportions of the delay in performing the first antenatal care

**FIGURE 2:**
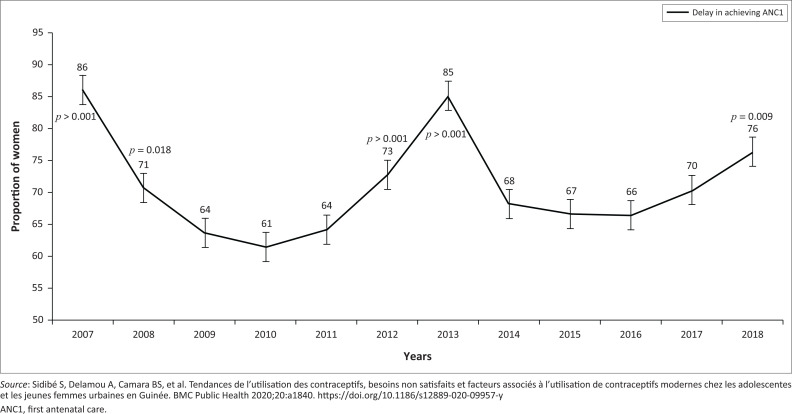
Annual trends in the proportion of women achieving late the first antenatal care in Guinea from 2007 to 2018.

Figure 2 shows the trends in the proportions of the delay in performing the ANC1 in Guinea from 2007 to 2018. A total of 14 546 women were included in the study; 10 619 women were late in performing the ANC1 between 2007 and 2018, for an overall proportion of 73%. Between 2007 and 2010, the proportion of the delay in the performance of the ANC1 experienced a decrease, from 86% (95% CI: 81–90) to a low of 61% (95% CI: 58–65) in 2010. Whereas between 2011 and 2013, we note a progressive increase from 64% (95% CI: 61–68) in 2011 to a peak of 85% (95% CI: 81–88). After this peak, a drop in the proportion was observed from 85% in 2013 to 68% (95% CI: 64–72) in 2014, and this decrease remained stable until 2016 before experiencing a progressive increase from 66% (95% CI: 63–70) in 2016 to 76% (95% CI: 72–80) in 2018. This proportion experienced a significant decrease between the periods 2007–2008 and 2008–2009, falling respectively from 86% to 71% (*p*-value < 0.001) and from 71% to 64% (*p*-value = 0.018). Between 2011 and 2012, the trend was an increase from 64% to 73% (*p*-value < 0.001) between the two periods before reaching a peak of 85% in 2013. However, a non-significant decrease was observed between 2013 and 2017 (*p*-value > 0.05). During the last period (2017–2018), the trend in the proportion of the delay in performing the ANC1 significantly increased from 70% in 2017 to 76% in 2018 (*p*-value < 0.001).

### Factors associated with the delay in performing the first antenatal care

In univariate analysis, the age of the woman, the region of residence, the place of residence, the level of education and that of her spouse, the wealth index, the marital status, the parity, the work of the woman, the distance from the health facility perceived as a problem by the woman, the profile of the health worker who received the woman for the first time, and the history of caesarean section were statistically significantly associated with the delay in performing the ANC1 (Table 2).

**TABLE 2:** Univariate and multivariate analyses of factors associated with delay in achieving first antenatal care in Guinea from 2007 to 2018.

Variables	Delay in achieving of first antenatal care					
	Crude OR	95% CI	*p*	Adjusted OR	95% CI	*p*
**Age group (years)**						
15–24	Ref.	Ref.	-	Ref.	Ref.	-
25–34	1.21	0.86–1.49	< 0.001	1.00	0.82–1.21	0.975
35–49	1.65	0.22–0.47	< 0.001	1.36*	1.08–1.69	0.008
**Region of residence**						
Labé	Ref.	Ref.	-	Ref.	Ref.	-
Boké	1.82	1.41–2.34	< 0.001	2.13*	1.58–2.87	< 0.001
Conakry	0.61	0.47–0.78	< 0.001	1.08*	0.77–1.51	0.642
Faranah	1.11	0.85–1.45	0.417	1.58*	1.19–2.11	0.002
Kankan	0.92	0.69–1.23	0.584	1.38*	1.07–1.77	0.011
Kindia	1.01	0.78–1.31	0.892	0.87	0.67–1.12	0.288
Mamou	1.09	0.84–1.42	0.477	1.34*	0.98–1.83	0.063
Nzérékoré	2.29	1.61–3.24	< 0.001	4.97*	3.58–6.91	< 0.001
**Place of residence**						
Urban	Ref.	Ref.	-	Ref.	Ref.	-
Rural	1.67	1.44–1.93	< 0.001	0.76	0.59–0.99	0.048
**Household wealth index**						
Rich	Ref.	Ref.	-	Ref.	Ref.	-
Poor	1.87	1.64–2.13	< 0.001	1.76*	1.39–2.22	< 0.001
Average poor	2.15	1.84–2.50	< 0.001	1.72*	1.34–2.23	< 0.001
**Woman’s level of education**						
No formal education	1.95	1.48–2.58	< 0.001	2.38*	1.05–5.43	0.039
Primary	1.14	0.84–1.54	0.380	2.04	0.88–4.73	0.095
Secondary	1.04	0.79–1.37	0.734	1.35	0.88–2.05	0.157
Higher	Ref.	Ref.	-	Ref.	Ref.	-
**Partner’s level of education**						
No formal education	1.61	1.43–1.82	< 0.001	1.17	0.94–1.45	0.165
Primary	1.17	0.97–1.40	0.103	0.86	0.61–1.24	0.435
Secondary	1.64	1.24–2.16	< 0.001	1.96*	1.22–3.15	0.005
Higher	Ref.	Ref.	-	Ref.	Ref.	-
**Marital status**						
Married	Ref.	Ref.	-	-	-	-
Single	0.82	0.65–1.03	0.094	1.46*	1.03–2.06	0.030
Divorced/widow	1.41	1.04–1.91	0.023	0.96	0.65–1.43	0.867
**Profile of the health worker who provided the first antenatal care**						
Midwife	Ref.	Ref.	-	Ref.	Ref.	-
Physician	1.48	1.31–1.69	< 0.001	0.73	0.59–0.89	0.003
Technical health agent	0.65	0.58–0.74	< 0.001	0.91	0.75–1.10	0.331
Other†	7.57	6.848–0.39	< 0.001	3.97*	3.08–5.09	< 0.001
**Whether the pregnancy was intended**						
Yes	Ref.	Ref.	-	-	-	-
No	1.06	0.94–1.20	0.311	1.08	0.88–1.32	0.462
**Parity†**						
Primiparous	0.71	0.65–0.79	< 0.001	1.17	0.91–1.50	0.210
Multiparous	0.76	0.69–0.82	< 0.001	1.01	0.84–1.19	0.942
Large multiparous	Ref.	Ref.	-	Ref.	Ref.	-
**History of caesarean section**						
No	Ref.	Ref.	-	Ref.	Ref.	-
Yes	0.71	0.56–0.91	0.007	1.05	0.68–1.62	0. 821
**Whether the woman had a job**						
No	Ref.	Ref.	-	-	-	-
Yes	1.23	1.11–1.37	< 0.001	-	-	-
**Decision making to go for antenatal care**						
Woman alone	Ref.	Ref.	-	Ref.	Ref.	-
Woman and her partner	0.92	0.75–1.14	0.489	1.14	0.88–1.45	0.318
Partner alone	0.84	0.68–1.04	0.113	1.22	0.96–1.54	0.098
**Distance to the health facility perceived as a problem**						
No	Ref.	Ref.	-	Ref.	Ref.	-
Yes	1.30	1.12–1.51	0.001	0.99	0.85–1.16	0.953

Ref., reference group; CI, confidence interval; OR, odds ratio (rating report).

* , Significant at the 5% level.

† , Other includes: traditional birth attendant; community or village health worker; relatives/friends/neighbours; relatives/friends/neighbours.

In the multivariate analysis, only the age of the woman, the region of residence, the level of education and that of her spouse, the wealth index, the place of residence, the marital status, the status of the health agent who received the woman for the first time were independently associated with the delay in performing the ANC1. Women aged 35–49 years compared to those aged 15–24 years had 1.36 times the risk of being late for the ANC1 (AOR: 1.36; 95% CI: 1.08–1.69). Women living in the regions of Boké (AOR: 2.13; 95% CI: 1.58–2.87), Faranah (AOR: 1.58; 95% CI: 1.19–2.11), Kankan (AOR: 1.38; 95% CI: 1.07–1.77), Nzérékoré (AOR: 4.97; 95% CI: 3.58–6.91) were more likely to be late for the ANC1 compared to women in the region of Labé. Poor (AOR: 1.87; 95% CI: 1.64–2.13) and middle-poor (AOR: 2.15; 95% CI: 1.84–2.50) women compared with rich women were 1.87 and 2.15 times more likely to be late for the ANC1, respectively. Women with no education level compared with those with higher education were 2.38 times more likely to be late for the ANC1 (AOR: 2.38; 95% CI: 1.05–5.43). Single women compared with married women were 1.46 times more likely to be late for the ANC1 (AOR: 1.46; 95% CI: 1.03–2.06). In contrast, women who resided in rural areas compared to those in urban areas had a 24% reduction in the risk of being late for the first ANC1 (AOR: 0.76; 95% CI: 0.59–0.99).

## Discussion

This study shows that in Guinea between 2007 and 2018, more than two-thirds (73%) of women with at least one live birth were late for the ANC1. The trend in this delay decreased between 2007 and 2010 from 86% to 61% and between 2013 and 2016 from 85% to 61%, then increased between 2010 and 2013 from 61% to 85% and between 2016 and 2018 from 66% to 76%. The factors associated with the delay in performing the ANC1 were the woman’s age, the region of residence, the woman’s and her spouse’s level of education, the wealth index, the area of residence, and the status of the agent who received the woman for the first time. These different results have important implications for public health policies and practices in Guinea. This study reveals that 73% of women with at least one live birth were late for the ANC1 between 2007 and 2018. Our results are higher than those found by Alamneh et al. in Ethiopia in 2016.^9^ This dissimilarity could be related to the difference in the definitions of delay. In the Ethiopian context, the delay in performing the ANC1 is after the 16th week of pregnancy unlike in Guinea where the delay is considered after the 12th week of pregnancy.

Our study highlights the reduction in the proportion of delays between 2007 and 2010 and between 2013 and 2016 as well as an increase in this proportion between 2010 and 2013 and between 2016 and 2018. This finding differs from those of Alamneh et al. in Ethiopia and Fagbamigbe et al. in Nigeria according to which the proportion of women who have completed the ANC1 has decreased over time.^9,10^ The possible reason for the fluctuation in the trend of this proportion in the Guinean context is the compromise of efforts to improve maternal and child health. The decrease in the proportions of delays between 2013 and 2016 could be explained by the effect of the free policy and awareness-raising activities, mobilisation by the community health workers and the community relays within the community. The increase in the proportion of the delay between 2016 and 2018 corresponds to the post-Ebola period in Guinea. The possible reason could be the relaxation of awareness-raising and community mobilisation activities by community health workers and community relays following the withdrawal of the financial partners in the process of strengthening the health system. This situation would contribute to the increase in obstetric complications, maternal and neonatal deaths. Based on these results, we recommend ensuring the sustainability of the achievements aimed at improving maternal and child health in Guinea through the transfer of skills between partners and frontline workers.

According to this study, women in the age group of 35–49 years were at higher risk of late ANC1 compared to other groups of women. This observation is consistent with the results of a systematic review^23^ and that of Girma et al. in Ethiopia.^12^ The reason why women aged 35–49 years delay their first ANC could be that they have already experienced uncomplicated pregnancies and may feel more confident in their previous experience of prenatal care, and are less afraid than younger women because of insufficient information on the disadvantages of late ANC. Quality awareness could encourage women to seek prenatal care early in pregnancy.

The results revealed that women living in poor and middle-poor households were at increased risk of late onset of the ANC. Previous research conducted by Mlandu et al. in three African countries (Democratic Republic of the Congo, Kenya and Tanzania) showed similar results.^24^ The prioritisation of other expenses to the detriment of transportation-related expenses could explain this delay among women from poor and middle-poor households in Guinea. It would be important to strengthen the advanced strategies by planning this activity in all isolated localities (villages, hamlets, countryside) which could help to cover transportation costs and ensure the effectiveness of the policy of free prenatal care in Guinea.

Women who lived in the regions of Boké, Faranah, Kankan and Nzérékoré were more likely to come late for the ANC1 compared to those in the region of Labé. This is consistent with the results of studies conducted in Nigeria^25^ and Ethiopia.^26^ Local cultural norms could be the reasons for the delay in the regions. It is essential to adapt health services and interventions to the socio-cultural norms of the locality.

This study reveals that women who lived in rural areas were less likely to come late for the ANC1 compared to those in urban areas. This finding is different from the results of studies conducted in Myanmar, Ethiopia and Nigeria.^25,26,27^ This difference could be explained by the fact that in the Guinean context, the activity of community health workers is much more accentuated in rural areas on one hand, and on the other hand, in rural areas there are no other alternatives to confirm pregnancy except in health structures.

### Limitations and strengths

This study has some limitations which must be taken into account in the interpretation of the results. It allows to establish an association between the delay in performing the ANC1 and the factors, but cannot establish a causal link because of its design (cross-sectional study). In addition, the dependence of this study on the exhaustiveness of the data on the variables of interest had excluded the evaluation of the factor access to the media because of insufficient data. However, it has strengths also. It examines the trends and the factors associated with these delays over a period of 10 years, using nationally representative data. This allows to generalise the results and to apply them to policies and interventions at the national level.

### Implications for the public health practices and research

The results of this research emphasise the need to adapt health services and interventions to the socio-cultural norms of the locality.

To strengthen advanced strategies by planning this activity in all isolated localities (villages, hamlets, countryside) which could help to cover transportation costs and ensure the effectiveness of the policy of free prenatal care in Guinea.

Also, to ensure the sustainability of the achievements aimed at improving maternal and child health in Guinea through the transfer of skills between partners and frontline agents.

## Conclusion

This study shows that in Guinea, between 2007 and 2018, 7 out of 10 women who had at least one live birth were late for the ANC1. The trend of this delay fluctuated every 3 years or 4 years. The advanced age of the woman, the region of residence, the level of education of the woman and that of her spouse, the wealth index, the area of residence, the status of the agent who received the woman for the first time were the factors associated with this delay. The strategies to be implemented should take these factors into account.

The strengthening of door-to-door awareness-raising activities by community relays and the effectiveness of the free prenatal care policy could encourage women to benefit from prenatal care from the beginning of pregnancy.
